# Temporal discrimination at the neck region is associated with severity of cervical dystonia

**DOI:** 10.1038/s41598-025-31139-2

**Published:** 2025-12-03

**Authors:** Thorsten M. Odorfer, Annika Junge, Jens Volkmann, Daniel Zeller

**Affiliations:** Department of Neurology, University of Würzburg, Josef-Schneider-Str. 11, 97080 Würzburg, Germany

**Keywords:** Cervical dystonia, Temporal discrimination, Proprioception, Sensorimotor integration, Neurophysiological endophenotyping, Diseases, Medical research, Neurology, Neuroscience

## Abstract

Cervical dystonia (CD) is a rare movement disorder marked by involuntary neck muscle contractions. Although somatosensory dysfunction has been proposed, the underlying pathophysiology remains unclear. Temporal discrimination (TD) deficits are discussed as potential markers of a dystonic endophenotype, possibly linked to proprioceptive impairments. This study aimed to assess TD in the neck region and explore its relationship with proprioception in head movements. We evaluated somatosensory temporal discrimination threshold (STDT) and temporal discrimination movement threshold (TDMT) in 20 CD patients and 20 healthy controls (HC). Both measures were applied directly to the cervical region: STDT via skin stimuli on the lateral neck and TDMT via muscle stimuli to the splenius capitis. Proprioception was assessed through a head rotation task. Clinical severity was measured using the TWSTRS scale. STDT and TDMT thresholds were significantly elevated in CD patients compared to HCs and correlated positively with dystonia severity. However, there were no group differences in proprioceptive performance, nor was proprioception correlated with TD or TWSTRS scores. TD is clearly altered in CD and associated with symptom severity, supporting its role in dystonia pathophysiology. In contrast, no proprioceptive deficits were observed, and no link between TD and proprioception was found. This suggests TD and proprioception may represent distinct dysfunctions rather than connected elements of sensorimotor integration. Further studies are needed to refine proprioceptive testing in CD and clarify its relationship with TD.

## Introduction

 As a clinical symptom, dystonia refers to an involuntary, sometimes painful, contraction of muscle groups. Beyond, the term dystonia describes a heterogeneous group of movement disorders where dystonic postures and/or movements are at the core of the clinical picture. These disorders are classified, inter alia, according to the location of the dystonic symptoms^1^. Cervical dystonia (CD) is the most common form of focal dystonia. Patients are clinically characterised by unnatural twisting of the neck and movements of the head. In addition, CD patients often suffer from non-motor symptoms such as depression, anxiety and social stigma. The burden of disease in this patient group can be significant^2^. Patients are primarily treated with selective denervation of the dystonic muscle groups by injection of botulinum neurotoxin (BoNT) or, less commonly, with anticholinergic drugs^3,4^. For patients who do not respond to these therapies, deep brain stimulation (DBS) of the internal globus pallidus is available as a second-line treatment^5,6^.

For a long time considered a disease of the basal ganglia, a body of research on the pathophysiology of dystonia now points to a network disorder of the central nervous system including cortical structures, the thalamus and the cerebellum^7,8^. From a neurophysiological perspective, deficits in three processes are considered to contribute to the development of dystonic phenotypes: altered cortical plasticity^9,10^, loss of cortical inhibition^11,12^, and altered sensorimotor integration^13,14^. In order to support these pathophysiological assumptions, there are increasing efforts to characterise stable biomarkers including the use of neurophysiological paradigms to define so-called endophenotypes. In this context, the term endophenotype refers to any measurable component potentially linking clinical presentation (phenotype) and its genetic basis (genotype).

One such paradigm is Temporal Discrimination (TD), which tests the cognitive ability to distinguish between stimuli presented in rapid succession. TD has already been described to be altered in several forms of dystonia, which is considered a possible correlate of altered sensorimotor integration in dystonia patients^15,16^. Specifically for CD, the paradigm has often been studied at the extremities^17–20^, whereas, to the best of our knowledge, only one study has assessed (somatosensory) TD at the neck region^21^. This appears surprising as the focal appearance draws natural interest in the physiology of this particular body region.

Assessing TD directly in the neck region offers several advantages: it targets the body region clinically affected by cervical dystonia, reduces potential confounding by peripheral impairments such as neuropathy or radiculopathy, allows the investigation of region-specific alterations, and enables the simultaneous assessment of proprioception in the dystonic region.

In a preliminary study, we discussed a possible association between impaired TD and reduced proprioception in healthy subjects and patients with polyneuropathy^22^. In dystonia, patients are known to exhibit proprioceptive deficits^23,24^, which may play a role in linking the endophenotypic significance of TD to the phenotypic presentation, together with other processes such as abnormal basal ganglia and cerebellar function.

Based on these considerations and preliminary examinations, we aimed to test the following three hypotheses: (i) multimodal assessment of TD at the neck region is feasible, (ii) TD at the neck region is reduced in people with CD compared to healthy controls, and (iii) TD is correlated with performance in a proprioception test of head movements.

## Materials and methods

The protocol conformed to the tenets of the Declaration of Helsinki and was approved by the Ethics Committee at the Medical Faculty of the University of Würzburg. All participants gave written informed consent to participate in the study.

### Participants

A total of 20 patients with CD, as diagnosed by a movement disorders specialist, were recruited from our outpatient clinic for movement disorders. Clinical assessment included the motor subscale of the Toronto Western Spasmodic Torticollis Rating Scale (TWSTRS)^25^. Demographic data and clinical characteristics of the patients are summarised in Table 1. Most patients were treated with periodic injections of BoNT A, and only a few received concomitant oral antidystonic therapy or other CNS-active medications (see Table 1 for details). In all cases, dosage had been stable for at least 3 months without subjective side effects. The experiment was conducted at least 10 weeks after the last injection of BoNT A, when both the investigator and the patient judged that there were no or only minor treatment effects remaining. The study excluded patients with clinically relevant psychiatric or neurological comorbidities. All participants were screened for cognitive impairment and depression (MoCA, BDI); only those with normal scores were included. In addition, a control group of 20 healthy volunteers (HC), matched for age and sex, was recruited. All subjects were assessed for clinical deficits in deep sensation and proprioception, testing both pallesthesia and positional sense of the hallux. Individuals with abnormalities were excluded as peripheral afferent dysfunction may interfere with TD assessment^22^.

**Table 1:** Demographic and clinical characteristics of cervical dystonia patients.

No.	Age	Sex	Disease duration (years)	TWSTRS motor score	BoNT treatment	Concomitant medication	Dominant Pattern	History of head tremor
1	73	f	35	16	x	Antidepressants	LC left	
2	44	m	18	17	x		LC left	
3	54	f	7	7	x		TC right	x
4	60	f	24	13	x		LC left	
5	72	m	50	21	x		DT	x
6	59	f	4	19	x	Anticholinergics	LC left	
7	61	m	16	18	x		TC right	x
8	56	m	12	7	x		TC right	x
9	64	f	2	21	x	Anticholinergics	TC left	
10	57	f	25	20			AC	x
11	42	f	18	18	x	Anticholinergics	TC right	x
12	70	f	26	17	x		TC left	
13	60	m	20	8	x	Anticholinergics	LC right	x
14	66	f	17	13		Anticholinergics	LS	x
15	65	f	30	22	x		AC	x
16	66	f	19	18	x		TC right	x
17	36	m	8	8	x		SS	
18	57	f	40	17			TC right	x
19	57	m	9	6	x	Anticholinergics	TC right	
20	62	f	15	20	x	Anticholinergics	TC right	

f = female, m = male, TWSTRS = Toronto Western Spasmodic Torticollis Rating Scale, BoNT = Botulinum-Neurotoxin, LC = Laterocollis, TC = Torticollis, DT = Dystonic Tremor, AT = Anterocollis, LS = Lateral Shift, SS = Sagittal Shift.

### Measures of temporal discrimination

**Fig. 1:**
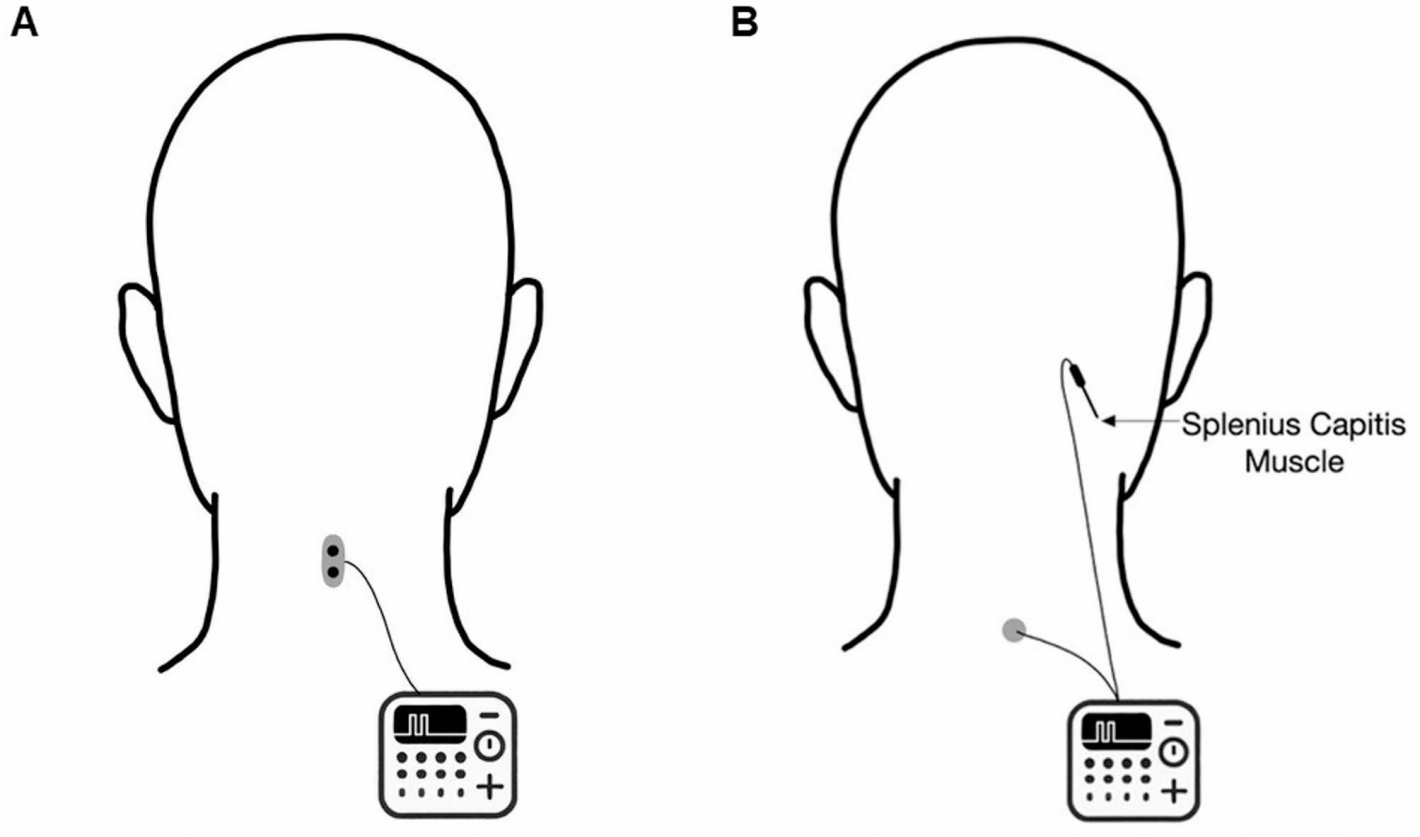
Schematic illustration of the temporal discrimination measurements: STDT (**A**) and TDMT (**B**).

Two variants of TD, i.e. somatosensory and kinaesthetic TD, were investigated in this study. The former tests the perception of superficial stimuli on the skin, while the latter refers to the perception of electrically induced muscle movements. Surface electrodes (anode and cathode 1 mm in diameter and 1.5 cm apart) were placed over the lateral neck region to test somatosensory TD thresholds (STDT) (see Fig. 1A). A constant current stimulator (Digitimer, Welwyn Garden City, United Kingdom) delivered pairs of square-wave electrical stimuli of 0.2 ms duration. Stimulation intensity was individually determined by gradually increasing current until participants could clearly perceive stimuli (i.e., 10 out of 10 trials). The stimuli were presented in ascending and descending sequences, each performed twice, starting either from 30 ms or 300 ms, with 5 ms increments. The average was taken for further analysis. STDT was defined as the shortest interstimulus interval (ISI) when participants perceived two separate pulses. For the assessment of kinaesthetic TD, the so-called TD motor threshold (TDMT) was determined. For this purpose, a monopolar electromyography (EMG) needle electrode was inserted into the right splenius capitis muscle (see Fig. 1B). The anode was a surface electrode which was placed above the C7 vertebral body. Using the same stimulation protocol as described above, the TDMT was defined as the shortest ISI at which subjects were able to discriminate clearly (i.e. three times in a row) two separate contractions of the stimulated muscle. Again, the mean of four attempts (i.e. two runs in both ascending and descending order) was used for further analysis.

### Proprioception task

**Fig. 2:**
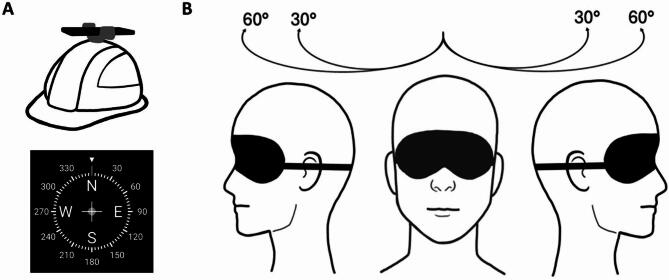
Schematic illustration of the proprioception task (modified head-to-target repositioning task): goniometer-based device using the iPhone compass application (**A**) and assessing predefined head rotations (**B**).

Proprioception was assessed via a modified head-to-target repositioning task^26,27^ using a goniometer-based device consisting of a helmet with a smartphone (iPhone 12 Pro, Apple, Los Altos (CA), USA) attached (see Fig. 2A). Head position angles were measured using the iPhone compass application^28–30^. During the test, great care was taken to ensure that the device was positioned horizontally, in order to avoid angular errors. Participants wore an eye mask and performed the tasks without visual feedback. At the start of the procedure, each participant was instructed to adopt a natural and relaxed head position that *subjectively* corresponded to a neutral gaze direction, without engaging in compensatory muscle activity to correct dystonia. This position was defined as the individual neutral position and referenced as 0 degrees in the compass application. CD patients and HC were made familiar with four different head positions corresponding to rotations of 30 and 60 degrees to the left and right, respectively (see Fig. 2B). The learning process was as follows: Starting from the neutral position, the experimenter passively rotated the participant’s head to the respective position, where it was held for two seconds. This procedure was repeated twice for each of the four positions. The ability to actively reproduce the learned head positions was then assessed. Each trial started from the neutral position, where initially the participant’s head was fixed by the experimenter. Then, the experimenter announced one of the four positions in a pseudorandomised order and released the participant’s head. The participant was instructed to actively move the head to the announced position. When the target position was subjectively reached, the participant signalled this by saying ‘yes’. Before the next position was announced, the experimenter repositioned the participant’s head back to the neutral position. Each position was announced a total of three times. Movements and angles were documented via screen recording of the compass application and subsequently analysed by the examiner. The absolute error between the target position and the achieved position was used as a measure of proprioceptive performance.

### Statistics

SPSS software (Version 29.0.2.0, IBM, Armonk (NY), USA, https://www.ibm.com/spss) was used for statistical analyses. According to the Shapiro-Wilk test, data were not normally distributed. Therefore, the Mann-Whitney U test was used for group comparisons and the Spearman test for correlations. Statistical significance was set at *p* < 0.05.

## Results

Twenty CD patients and 20 controls (HC) were included, with 13 women and seven men in each group. The mean age was 59.1 ± 9.6 years in the CD and 59.2 ± 9.9 years in the HC group (*p* = 0.961). In CD patients, the median TWSTRS motor score was 17 [range 6 to 22] points. The average disease duration was 19.8 ± 12.2 years. Eleven patients presented with dystonic head tremor (see Table 1).

**Fig. 3:**
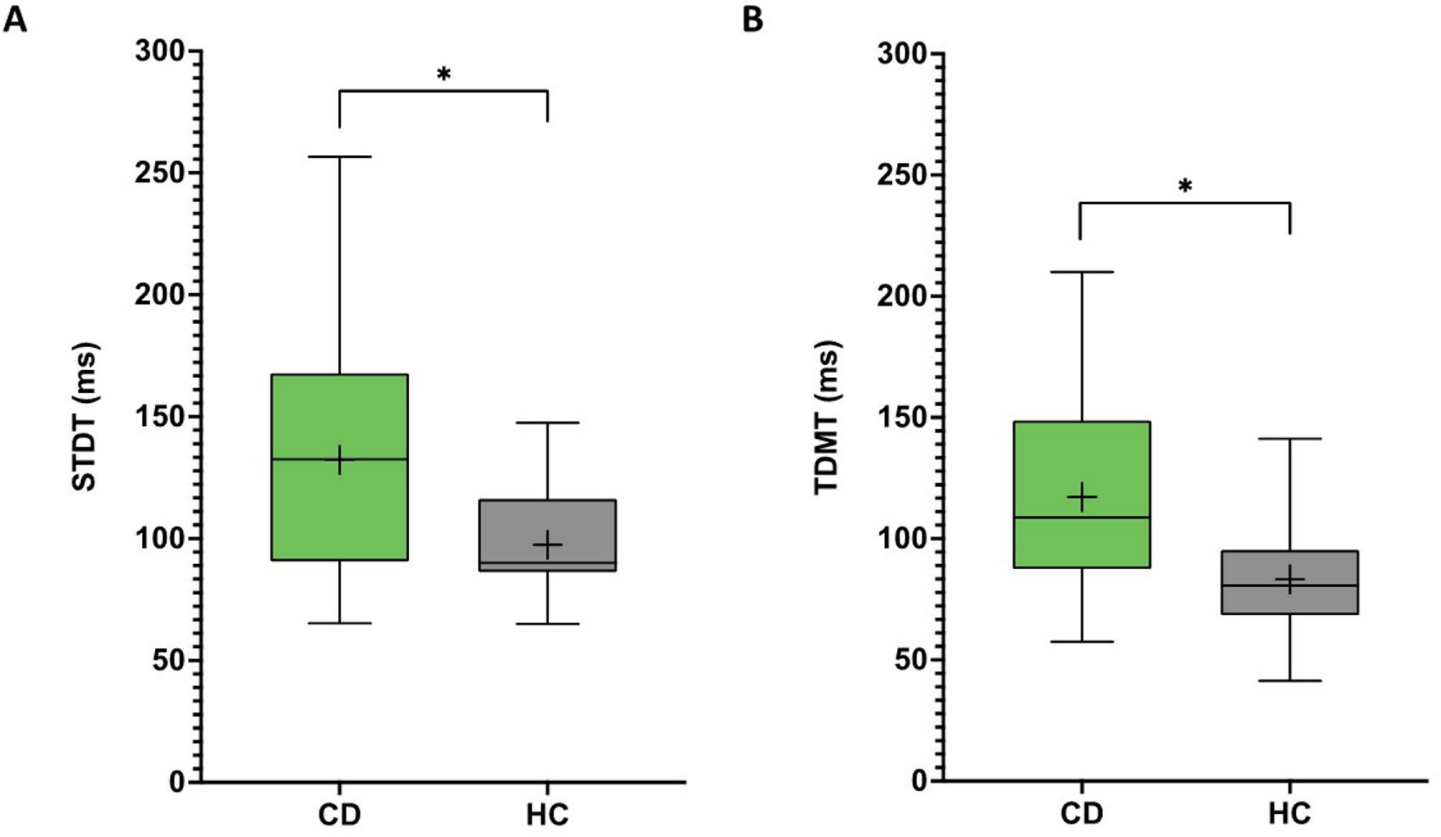
Somatosensory temporal discrimination threshold (STDT; **A**) and temporal discrimination motor threshold (TDMT; **B**) in cervical dystonia (CD) vs. healthy controls (HC). The asterisk indicates *p* < 0.05.

In both temporal discrimination paradigms, the threshold levels of the CD patients were significantly higher than those of the HC group: STDT CD group 132.2 ± 48.7 ms vs. STDT HC group 97.5 ± 21.6 ms (*p* = 0.029; Fig. 3A) and TDMT CD group 117.3 ± 42.6 ms vs. TDMT HC group 83.3 ± 22.7 ms (*p* = 0.017; Fig. 3B).

Regarding proprioceptive performance, patients with CD and HC showed comparable angular deviation from the target (9.6° ± 4.5° vs. 8.7° ± 4.0°, *p* = 0.659). A subgroup analysis comparing a CD cohort with (9.9 ± 5.3, *p* = 0.670) and without tremor (9.2 ± 3.7, *p* = 0.799) with HC also showed no group differences.

**Fig. 4:**
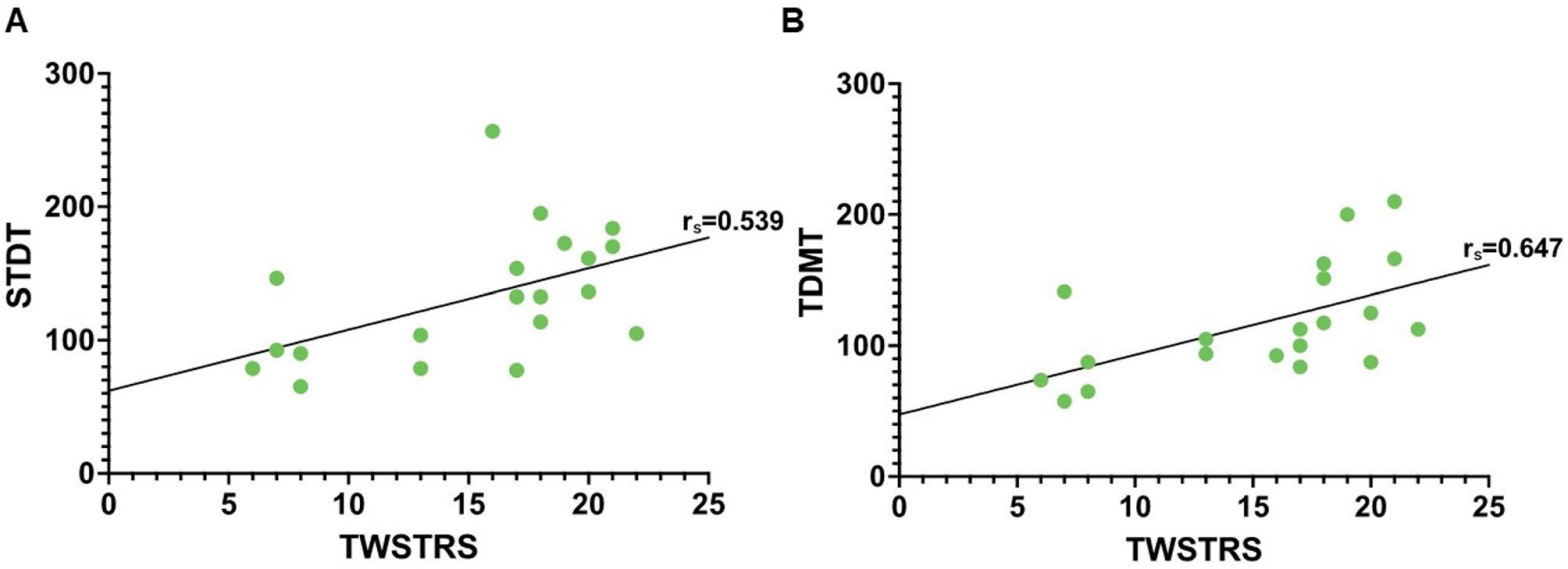
Correlation test of STDT (**A**) and TMDT (**B**) with dystonia clinical symptom severity (measured by TWSTRS).

Looking for associations of temporal discrimination performance with clinical and behavioral measures, respectively, higher STDT as well as TDMT levels were positively correlated with the severity of motor symptoms (i.e. higher TWSTRS scores) in CD patients: Spearman Rho STDT 0.539 (*p* = 0.014; Fig. 4A) and TDMT 0.647 (*p* = 0.002; Fig. 4B). In contrast, there were no significant correlations of the proprioceptive performance with clinical rating or TD performance.

As an additional finding, STDT performance correlated with age, i.e. older patients had higher thresholds (Spearman Rho 0.541, *p* = 0.014). However, this association was not observed for TDMT performance. Furthermore, STDT and TDMT did not differ between patients taking CNS-active medications and those who were not.

## Discussion

In this study, the temporal discrimination (TD) of somatosensory and kinaesthetic stimuli was assessed in the neck region of patients with cervical dystonia (CD) and matched healthy controls (HC). To the best of our knowledge, this is the first study to record TDMT from this region and to concomitantly assess physiological and behavioral measures of the affected body area in CD patients. We had hypothesized that (i) TD assessment in the neck region is feasible, (ii) TD levels are elevated in CD in comparison to HC and (iii) TD is correlated with pointing errors in a proprioception task involving the head. In brief: both STDT via surface electrodes and TMDT via EMG needle electrodes in the splenius capitis muscle proved technically feasible and produced plausible results, with STDT and TDMT significantly elevated in CD patients compared to HC. In addition, STDT and TDMT levels correlated significantly with the clinical symptom severity of cervical dystonia. However, there was no significant association of STDT or TDMT with proprioceptive performance in the plane of head rotation.

### Temporal discrimination and dystonia

Studies of STDT in dystonic patients were first published at the end of the last century and showed elevated thresholds in measurements from the hand affected by dystonia in patients with writer’s cramp^31^ and other non-focal dystonias that affect the upper limbs^32,33^. In the course of time, these findings were also extended to body regions not affected by dystonia – for example, to the non-affected hand in patients with hand dystonia^34,35^ or to the hand in patients with CD^36^ or blepharospasm^37^. It has even been shown that asymptomatic carriers of the DYT1-TOR1A mutation present with similar alterations^38^. In particular, the latter finding can be interpreted as a strong argument for considering elevated STDT levels as a dystonic endophenotype, i.e. a heritable trait that – albeit with incomplete penetrance - can reflect a predisposition for the development of clinical dystonia. For patients with CD, there are a number of other published observations on this topic: Elevated STDT also extends to the skin of the face and neck^21^, STDT alterations do not change with therapies such as BoNT^39^ or DBS^40^, and they do not change spontaneously over time^41^. Interestingly, however, increased STDTs could also be demonstrated in patients clinically classified as having psychogenic dystonia^42^. In conclusion, the finding of an altered STDT appears to be quite robust in the context of the previous literature, and our results explicitly confirm the preliminary findings of Scontrini et al. that STDT is also increased in the neck region of CD patients^21^.

Whereas the STDT tests the processing of somatosensory stimuli, the TDMT primarily tests the perception of kinesthesia^43^. Compared to STDT, much less data is available on this paradigm. Again, elevated thresholds have been described in focal dystonia, namely writer’s cramp^44^. Somewhat surprising, one study found TDMT normal in patients with dystonic tremor and elevated in patients with essential tremor, while exactly the opposite was described for STDT^45^. As to our knowledge, TDMT was recorded either from the flexor carpi radialis or the first dorsal interosseus muscle in all published studies. Overall, scientific evidence for TDMT in dystonia is less clear than for STDT.

The following conceptual hypothesis regarding the neuroanatomical and neurophysiological underpinnings of TD has emerged from the results of various neuroimaging studies (reviewed in^15^: TD involves a brainstem–cerebellar–basal ganglia network, with the superior colliculus acting as a key sensory input hub. The cerebellum contributes to precise timing and sensorimotor integration, supporting both somatosensory and kinesthetic processing^46^. Basal ganglia integrity is crucial for integrating sensory input from the superior colliculus, thalamus, and cerebellum^47^. Modulated by dopamine, this network helps to select stimuli appearing important for behaviour. Additionally, sensory signals reaching the S1 cortex refine perception by inhibitory interneurons, thereby enhancing TD^48,49^.

Based on such a neural circuit model, the extent to which deficits in TD may underlie the pathophysiology of CD can be speculated about: Dystonia as well as abnormal TD may stem from a common disruption in the midbrain network that controls attentional orienting. Impaired GABAergic inhibition in the superior colliculus causes abnormal burst firing in visual sensory neurons. This abnormal firing leads to prolonged neuron activation, which then induces hyperexcitability in premotor neurons in the deep layers of the superior colliculus. Along subsequent motor pathways, this hyperactivity may result in the muscle spasms seen in CD^48^. In addition, the cerebellum likely modulates temporal processing within this network, further influencing abnormal TD and motor output in dystonia.

Apart from the midbrain, the classic basal ganglia structures certainly play a role in explaining the link between TD and dystonia. In focal dystonia, structural and functional putaminal alterations have been described. Putaminal enlargement is seen in adult-onset dystonias like blepharospasm^50^ and is linked to abnormal TD in laryngeal dystonia^51^. The putamen is involved at early stages of the TD sequence and is activated during stimulus differentiation^52^, suggesting its enlargement reflects dysfunction in both focal dystonia and temporal processing^15^.

The observed correlation between TD abnormalities and clinical disease severity in CD may seem at odds with the idea of TD as an endophenotypic marker—that is, a heritable trait largely independent of symptom expression. Tremor could be a potential contributor, but in our cohort, TD thresholds did not differ between patients with and without tremor, making this unlikely. Instead, elevated TD may rather serve as a biomarker of network dysfunction within the basal ganglia–cerebellar–brainstem circuitry, rather than a purely stable endophenotypic trait.

This perspective aligns with ongoing debates about the stability of TD as an endophenotype and highlights the need to consider broader cognitive and non-motor contributions to dystonia. It has been hypothesised that not only TD, but the decision-making process itself is retarded in dystonia patients^53^. This seems justified given the high prevalence of non-motor symptoms such as depression, anxiety, and cognitive deficits in dystonia^54^. Of note, however, in a preliminary study addressing this potential confounding factor, we found equal reaction times in CD patients and healthy controls in a simple reaction time paradigm^55^.

### Proprioception and dystonia

The presence of proprioceptive deficits in focal dystonia is widely accepted by the scientific community and has been substantiated by a body of evidence (reviewed in^23,56^. The deficits in TD and proprioception are each considered to be independent correlates of impaired sensorimotor integration in dystonia (c.f^57^. for a review). Abnormal processing of sensory information is considered to be in the centre of pathophysiology, resulting in dystonic motor output.

Sensorimotor integration involves the sensory and motor cortices, as well as premotor areas, the basal ganglia and the cerebellum^58^. Proprioceptive signals reach the somatosensory cortex via the dorsal column–medial lemniscus pathway passing thalamus and the cerebellum via spinocerebellar tracts for coordination and balance. Obviously, there is a significant overlap between TD and proprioception in terms of their shared networks. Recent work has also shown that basal ganglia structures are involved in the processing of proprioceptive stimuli by recording local field potentials in the pallidum of dystonia patients with DBS^59–61^.

Proprioceptive deficits in CD have been described repeatedly. For example, the tonic vibration reflex has been found abnormal in dystonic patients on conventional upper limb testing^62,63^. Moreover, people with CD showed alterations in head alignment when the vibration test is applied to the neck muscles compared to HC^64^. Vibro-tactile stimulation procedures are currently even investigated as a therapeutic method^65–67^. Kinematic studies of purposeful arm movements in patients with CD showed discrete abnormalities, with movements being less accurate in CD than those in matched controls^68,69^. Furthermore, compared to controls, CD patients showed reduced capacity in anticipating the timing of human movements, but not those of objects^70^. Another study has demonstrated deficits in motion perception in CD patients using a motion platform^71^. Thus, there is remarkably little evidence of proprioceptive deficits in CD in the dystonic body region. This may simply be due to the fact that proprioception testing of neck and head movements is not trivial.

In an approach similar to ours, Avanzino et al. observed proprioceptive deficits at neck and upper limb muscles only in CD patients presenting with dystonic tremor^72^. In the same trial STDT of the hands did not correlate with proprioceptive performance of the upper limb, while TD at the neck region was not tested^72^. Contrary to those findings, we could not demonstrate behavioral proprioceptive differences at all, neither between CD patients and HC, nor beween CD patients with and without tremor. However, this could also be due to methodological differences: While our subjects were asked to turn their head from a neutral position to a previously learned target position, the task in the study of Avanzino and colleagues was to return to the neutral position after a passive head movement. Nevertheless, our study showed that TD and proprioceptive performance do not correlate, even when assessed in the body region affected by CD. This suggests proprioception and TD more likely to be independent variables and does not support our hypothesis of a direct association between defective TD and impaired proprioception. However, it is conceivable that abnormalities in sensorimotor integration, rather than peripheral proprioceptive input itself, are critical, possibly involving a broader brainstem–cerebellar–basal ganglia network.

### Limitations of the study

As common for studies in rare disease, the validity of the results is limited by the relatively small number of patients.

Methodologically, to increase comparability, we used the right splenius capitis muscle for TDMT in all patients, even though it was not the muscle most affected by dystonia in several patients. Individual selection of the muscle depending on clinical presentation would have been an alternative approach worth considering for future studies.

Assessment of proprioceptive performance was based on active pointing rather than position estimation. It cannot be excluded that sensitivity of this test was insufficient to detect subclinical deficits in the perception of head position. However, the approach was chosen for two reasons: First, we had found active pointing to be positively associated with TD performance in an earlier study^22^. Second, movement of the head by the experimenter might have introduced a bias due to tactile input, with possible effects similar to a sensory trick. Regarding the latter, however, our test apparatus comprising a smartphone mounted on a helmet may have represented a sensory bias itself. In future, non-contacting technical options like vision-based motion analysis should be applied.

Clinical symptoms were assessed using the TWSTRS motor score. However, this score does not include an item related to tremor which could have been used to correlate tremor severity with TD and proprioceptive performance. Given the influence of tremor on TD and proprioception identified in earlier studies^45,72^, the use of a scale capturing tremor may have been useful in disentangling the contribution of TD and proprioception to the dystonic tremor phenotype.

## Conclusion

The present study provides further evidence that TD is altered in CD, with elevated thresholds for STDT and TDMT. Notably, TD thresholds are positively correlated with motor symptom severity of dystonia. These findings suggest that TD may serve as a biomarker of network dysfunction underlying cervical dystonia, rather than a stable endophenotypic trait. In contrast to one of our hypotheses, our proprioceptive assessment of head positions did not show differences between CD and HC, and, though assessed in the dystonic body region, was not associated with TD thresholds. Thus, we were not able to establish impaired proprioception as a behavioral correlate of TD dysfunction, although both functions are known to activate an overlapping neural network and appear to be established markers of impaired sensorimotor integration, a core component in the pathophysiology of dystonia. Future studies will need to elucidate further aspects of proprioception in CD, especially in the neck region, e.g. by application of video-based methods. Understanding the interplay of TD, proprioception and clinical phenotype will ultimately pave the way for a comprehensive pathophysiological model of dystonia.

## Data Availability

The datasets generated during and/or analysed during the current study are available from the corresponding author on reasonable request.

## Abbreviations

BoNT: Botulinum neurotoxin
CD: Cervical dystonia
DBS: Deep brain stimulation
HC: Healthy controls
ISI: Interstimulus intervals
STDT: Somatosensory temporal discrimation threshold
TD: Temporal discrimination
TDMT: Temporal discrimination movement threshold
TWSTRS: Toronto Western Spasmodic Torticollis Rating Scale
